# Management and Treatment of Patients with Chronic Hepatitis B: Towards Personalized Medicine

**DOI:** 10.3390/v14040701

**Published:** 2022-03-28

**Authors:** Piero Colombatto, Barbara Coco, Ferruccio Bonino, Maurizia R. Brunetto

**Affiliations:** 1 Hepatology Unit and Laboratory of Molecular Genetics and Pathology of Hepatitis Viruses, Reference Center of the Tuscany Region for Chronic Liver Disease and Cancer, Department of Medical Specialties, University Hospital of Pisa, Via Paradisa 2, 56124 Pisa, Italy; p.colombatto@ao-pisa.toscana.it (P.C.); b.coco@ao-pisa.toscana.it (B.C.); 2 Institute of Biostructure and Bioimaging, National Research Council, Via De Amicis 95, 80145 Naples, Italy; ferruccio.bonino@unipi.it; 3 Internal Medicine, Department of Clinical and Experimental Medicine, University of Pisa, Via Savi 10, 56127 Pisa, Italy

**Keywords:** HBV, chronic hepatitis B, Interferon-α, nucleos(t)ide analogues, antiviral treatment, HBV functional cure

## Abstract

The currently available antiviral treatments (Peg-Interferon-α and Nucleos(t)ide Analogues, NA) for chronic hepatitis B (CHB) achieve a functional cure (serum HBsAg and HDV-DNA clearance) of HBV infection in a limited number of patients. Nevertheless, the continuous pharmacological suppression of viral replication by NA halts liver disease progression lowering the risk of HCC development and improving the survival. In the near future, to fully exploit the potential of old and new drugs for HBV treatment a personalized approach to the patients will be required according to an accurate definition of their virologic, immunologic and clinical profile.

## 1. Introduction

Hepatitis B virus (HBV) is not directly cytopathic and chronic Hepatitis B (CHB) results from persistence of a defective host’s immune response unable to control the viral infection. The majority (about 60%) of HBsAg positive individuals achieve spontaneously an effective control of viral replication with clearance of intrahepatic necro-inflammation and transition to HBeAg negative infection and eventually clearance of circulating HBsAg [1]. Nevertheless, even after HBsAg loss, HBV persists within some hepatocytes in form of viral mini-chromosome, supercoiled covalently closed HBV-DNA (cccDNA), which eventually triggers the reactivation of viral replication in case of impairment of immune competence [1]. Thus, the course of HBV infection is driven by a complex interplay between virus and host immune system whose dynamic equilibrium is responsible not only for the different phases of chronic HBV infection, but also affects the outcome of antiviral treatment. Accordingly, the response rate to therapy is influenced by the phase of infection (HBeAg positive or HBeAg negative/anti-HBe positive), viral load and liver disease activity. 

**Figure 1:**
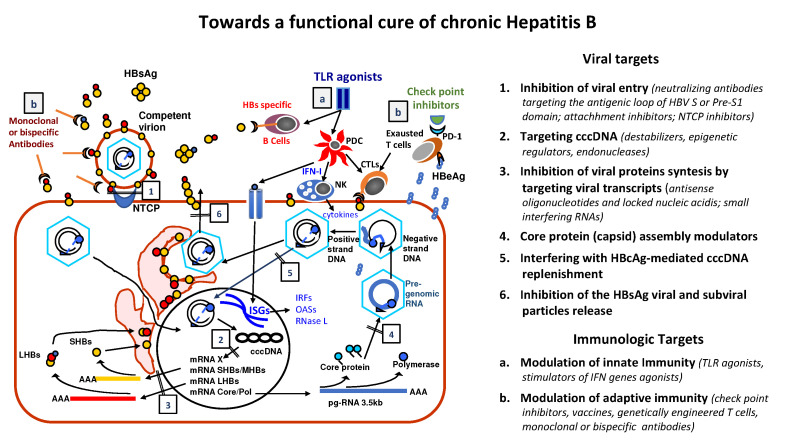
HBV replicative cycle and targets of the drugs under development to cure chronic hepatitis B patients. Description of their mode of action at the different steps of the HBV cycle or according to their modulation of immune response: (1) inhibition of viral entry by Na-Taurocholate Cotransporting Polypeptide (NTCP) receptor inhibitors or by neutralizing antibodies targeting the Pre-S1 domain of the HBsAg (b); (2) destabilization/degradation of the nuclear covalently closed circular DNA (cccDNA) or inhibition of its transcription; (3) inhibition of viral proteins synthesis by targeting viral mRNAs coding for HBx, small, middle and large (S, M and L) HBs, Core and Polymerase (Pol) proteins; (4) interference with Core protein assembly and (5) HBcAg-mediated cccDNA replenishment; (6) inhibition of viral and subviral particles HBsAg release; (a) modulation of innate immunity by Toll Like Receptors (TLR) agonists, which can activate Plasmacytoid Dendritic Cells (PDC), Natural Killer (NK) cells, or Interferon (IFN) Sensitive Genes (ISGs) response, which increase the production of several IFN Response Factors (IRFs) with broad-spectrum antiviral activity, similar to that of 2′-5′-oligoadenylate synthetase (OASs) and RNase L; (b) modulation of the adaptive immunity, by check point inhibitors acting on the Programmed Death 1 (PD-1) receptor of viral antigen-specific T cells exhausted because of the antigen overload, thus improving Cytotoxic T Lymphocytes (CTL) activity. Alternative approaches to increase recognition and elimination of HBV infected cells include genetically engineered T cells and therapeutic vaccines.

Current antiviral therapy is aimed to prevent the progression of CHB to cirrhosis and in cirrhotics to avoid or delay end-stage complications of liver disease and hepatocellular carcinoma (HCC) [1]. To this purpose two major therapeutic approaches are used: (a) to shift the host-virus equilibrium from pathogenic to non-pathogenic with a limited treatment course able to induce a sustained off-therapy control of HBV replication or (b) to suppress continuously viral replication. Interferon-α (IFN-α), is the major player of the former strategy and because of its side effects, is recommended in CHB patients without or with early cirrhosis. Instead, a long term, eventually lifelong, treatment with nucleos(t)ide analogues (NA) is used to inhibit viral replication in naïve patients with advanced or de-compensated cirrhosis or after IFN-α failure [1,2,3,4]. At present, there is great expectation for a significant improvement of CHB treatment with new compounds under development. Different drugs were designed to induce a more effective control of HBV infection by reducing permanently the number of infected cells with productive HBV infection or silencing viral cccDNA and/or stimulating HBV-specific host immune responses mimicking the spontaneous control of HBV infection (Figure 1) [5]. The premise is that future therapeutic strategies, eventually combining new and old drugs in a personalized manner, could lead to a permanent off therapy control of HBV infection with the clearance of both serum HBsAg and HBV-DNA (functional cure) in a much higher proportion of patients [5].

## 2. Currently Available Treatments

### 2.1. Interferon

**Figure 2:**
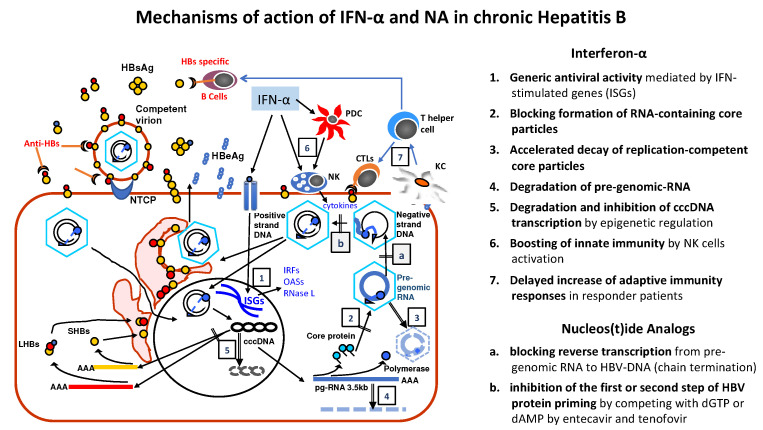
Interferon-α (IFN-α) has been shown to target different steps of HBV cycle either by generic antiviral mechanisms (1), via activation of the IFN Sensitive Genes (ISGs), or by specific blocking (2) and degrading (3) replication competent core particles, as well as pre-genomic-RNA (4) and nuclear covalently closed circular DNA (cccDNA), or by inhibition of cccDNA transcription (5). The main immune-modulatory activity of IFN-α consists in boosting the innate immune response (6) by stimulating Natural Killer (NK) cells, whereas IFN-α appears unable to restore the effector function of the HBV specific cytotoxic T cells (CTLs). Nevertheless, a late improvement of the adaptive immune responses (7), involving the functions of Kupfer Cells (KC), T Helper lymphocytes and Plasmacytoid Dendritic Cells (PDC), is observed in responder patients.

Interferon-α has multiple mechanisms of action including antiviral, anti-proliferative and immune-modulatory activities (Figure 2), however the exact mechanisms favoring the control of HBV infection are not yet completely understood [6,7]. In addition to its generic antiviral activity, that is mediated by cellular genes (IFN-stimulated genes, ISGs) which activate different pathways of antiviral defense in both infected and non-infected cells, IFN-α has been shown to inhibit directly HBV [8]. Multiple mechanisms are involved in this specific activity: blocking of the formation of RNA-containing core particles, accelerated decay of replication-competent core particles, degradation of both pre-genomic-RNA [9,10,11] and covalently closed circular DNA (cccDNA) mediated by the APOBEC3 family cytidine deaminase A3A [12]. Furthermore, IFN-α inhibits both HBV transcription and replication targeting directly the epigenetic regulation of cccDNA [13]. Thus, the overall antiviral activity of IFN-α could play a pivotal role in the control of HBV infection. Conversely, the role of IFN-α modulation of innate and adaptive immune-response remains to be clarified, particularly the effect on virus specific-CD8 cells [14], which show vigorous and multi-specific activation in subjects who achieved, either a spontaneous or after treatment control of HBV infection [15]. Available data suggest that IFN-α boosts the innate immune response, without a direct restoration of the effector function of the HBV specific-CD8 cell response [16,17]. However, the CD56^bright^ NK cells activation could favor antiviral mechanisms, which may unbalance the virus-host equilibrium as a pre-requisite for a later restoration of competent adaptive immune response. Accordingly, a stronger increase of NK cell TRAIL expression was associated with a greater reduction of viral load and HBsAg serum levels [17] and CD8 T cell responses were detected more frequently after therapy withdrawal in sustained responders [18]. 

Nucleos(t)ide Analogs (NA) block the RNA- and DNA-dependent DNA polymerase of HBV (a) and causes chain termination during reverse transcription of the pre-genome RNA (pg-RNA) into HBV-DNA. Entecavir and tenofovir also inhibit (b) the first or second step of HBV protein priming by competing with deoxyguanosine triphosphate (dGTP) or deoxyadenosine monophosphate (dAMP). 

At present, the reasons for the relatively low proportion of sustained responders remains to be explained, as well as the role of HBV genotype that strongly influences the response to interferon in vivo and in vitro [1]. Accordingly, several studies were addressed to investigate the role of genetic polymorphism in the individual susceptibility to IFN-α. In spite of some preliminary interesting data, to date final evidences supporting their use in clinical practice are missing. At variance with HCV, interleukin 28B gene polymorphism was not proven to influence the response rates in the setting of CHB [19]. Similarly, human leukocyte antigens (HLAs) that were found to be important determinants of HBV pathogenesis in Asian populations [20], did not show any significant correlation with IFN response in some studies [21], although some variants in the HLA-DPA1 region were found to correlate in other studies. However, the DPA1 rs3077 GG genotype, that correlated with on-therapy HBeAg/anti-HBe seroconversion in Asian patients, in Caucasian patients correlated with off-therapy response only in combination with the DPB1 (rs9277535) G-allele [22,23]. Growing evidences suggest that the IFN signalling pathway is subjected to genetic variability: among the molecules involved, the rs4845384 polymorphism of adenosine deaminases acting on RNA-1 (ADAR1), was associated with higher rates of spontaneous (OR = 1.36, 95% CI = 1.03–1.78, *p* = 0.028) and interferon induced (OR = 1.83, 95% CI = 1.13–2.96, *p* = 0.013) HBsAg sero-clearance in 725 HBV infected subjects [24]. Another polymorphism involving the signal transducer and activator of transcription 4 (STAT4), a member of the STAT family that localizes to the cytoplasm and is activated by different cytokines through the Janus kinase (JAK)-STAT signalling pathway, has been proposed as a new predictor of the response to IFNα therapy for HBeAg-positive CHB patients [25], as the rs7574865 GG genotype was associated with an half response rate, compared to the GT/TT genotype (19.3% vs. 39.1%, *p* = 4.15 × 10^−6^), in 466 Chinese patients. Overall, such preliminary results on genetic polymorphisms and IFN response, as well as the relevance of key and regulatory viral genes require further investigation [26,27]. In addition, probabilistic data provided by associative statistics do not impact in the single patient decision-making where only deterministic data and significant changes over time of therapy response indicators can pragmatically provide useful means for treatment modifications. Therefore, only a better understanding of the complex interactions between HBV and both the direct antiviral and immune modulatory activities of IFN-α will lead to the optimization of its use in individual patients and contribute to develop new therapeutic strategies with the new drugs.

#### 2.1.1. Efficacy of IFN-α Treatment

Standard IFN-α was the first treatment option for CHB and showed antiviral activity in both HBeAg positive and negative CHB patients [28,29]: serum HBV-DNA became undetectable (<1–10 pg/mL) in 37–56% of HBeAg positive CHB patients with 33% HBeAg loss, about 70% ALT normalization at the end of 4–6 months therapy course, over 80% of HBeAg to anti-HBe seroconversions maintained for 4–9 years after treatment discontinuation and up to 65% HBsAg loss in sustained responders [28]. The same treatment schedules (5–10 MU every other day for 16–24 weeks) when used in HBeAg negative/anti-HBe positive CHB were associated with high relapse rates (70–90%) in spite of good (70%) on treatment response. Thus, longer treatment courses (12–24 months) were attempted in HBeAg negative/anti-HBe positive CHB with higher sustained response rate (22–30% vs. 10–15%) [29] and 32–67% HBsAg loss in sustained responders within 4–7 years post-treatment [29,30,31,32]. 

In the last 15 years standard IFN-α was substituted by pegylated formulations whose active drug conjugated with polyethylene glycol (Peg) molecules has a prolonged half-life and can be administered once weekly. There were two formulations of pegylated interferon (Peg-IFN) available: Peg-IFN alfa-2b linked to a linear Peg molecule of 12 kD and Peg-IFN alfa-2a linked to a larger branched Peg molecule of 40 kD. Only the latter is authorized in CHB in Western Countries [33]. The virologic and biochemical response rates at the end of therapy of a 12-month course Peg-IFN-α in both HBeAg positive and negative patients are reported in Table 1. 

In HBeAg positive patients at 6–12 months post-treatment HBeAg to anti-HBe seroconversion was achieved in 29–32% of HBeAg positive [34,35,36]. In HBeAg negative CHB patients ALT normalization and HBV-DNA <2000 IU/mL at 6 months follow-up were observed in 38% of cases, overall, the response was maintained in 25% of patients up to 5 years with a 12% HBsAg clearance rate [37]. Therefore, IFN-α treatment may indeed induce a functional cure even if a limited number of cases: worthy to note that the overall rate of HBsAg loss in treated patients is much higher than achieved spontaneously in HBeAg negative infection [38]. 

Several cohort studies showed that sustained response to IFN-α is associated with a significant reduction of progression of chronic hepatitis to cirrhosis, end stage complications and longer survival [32,33,39]. IFN-α response reduces significantly the mortality rate in patients with cirrhosis, but it does not eliminate their HCC risk. Meta-analyses demonstrated that 4.6% of cirrhotic patients treated with interferon developed HCC vs. 9% of controls (*p* = 0.006) with a more significant reduction in those with cirrhosis in an earlier stage [40,41]. Available data show a better impact of Peg-IFN-α on HCC than NA, at least in Asian patients [42].

#### 2.1.2. Interferon Treatment Tailoring

Given the small proportion of sustained responders to Peg-IFN-α (20–30%), the identification of patients with higher chance of response or an early identification of non-responders would ensure a more cost/effective therapy. Baseline factors (viral genotype, sex and age, HBV DNA, HBsAg and ALT levels) were shown to correlate with response [1], however viral genotype, sex or age in spite of the highly significant statistical association with SVR in population studies, do not have the high predictive value required to guide treatment decision in the single patient. Thus, their use in combination with on-treatment predictors, such as HBsAg, HBcrAg or HBV-RNA, is under investigation to develop more personalized treatment scores. According to recent reports, an early serum HBV-RNA decline appears to correlate with response to Peg-IFN-α treatment, however only the combined kinetics of viral nucleic acids (HBV-DNA and HBV-RNA) and antigens (HBsAg and HBcrAg) identifies with higher accuracy the patients who achieve a functional cure [43,44].

In addition, the evidence that lower baseline HBV DNA levels (<10^7^ IU/mL) combined with higher ALT levels (>3 times upper limit of normal) correlated with higher response rates in both HBeAg-positive and HBeAg-negative CHB suggest to start treatment during such a phase of the highly “dynamic” interplay between HBV and host’s immune system [45]. Accordingly, in HBeAg-negative CHB the frequently elapsing hepatitis exacerbations could represent the most appropriate time to start IFN-α treatment, as confirmed by the evidence that also high baseline IgM hepatitis B core antibody (IgM anti-HBc) levels associated with a significantly higher response rate [46]. 

Currently, serum HBsAg kinetics represents an important guide for Peg-IFN-α therapy predicting the on-treatment response. In HBeAg negative patients the extent of HBsAg decline from baseline to the end of treatment correlated with the 3 years post-treatment HBsAg loss and 10 IU/mL HBsAg levels at end of treatment associated with 52% HBsAg clearance probability at 3 years post-treatment follow-up, compared to only 2% in patients with higher levels [47]. The end-of-therapy (EOT) predictive value of HBsAg levels was higher than that of HBV DNA since HBsAg clearance was achieved only in 15% of the patients with undetectable HBV DNA at week 48. Similarly, the most pronounced serum HBsAg declines occurred in HBeAg positive responders to Peg-IFN-α, as compared to small or absent reductions in non-responders [48]. A major limitation of the widespread use of HBsAg is the need-to-know HBV genotype, which influences both baseline serum levels and treatment kinetics of HBsAg. In HBeAg negative CHB patients the greatest difference in HBsAg serum levels between responders and non-responders was observed between weeks 12 and 24 in genotype A patients and between baseline and week 12 in genotypes B and D; whereas in genotype C patients it was impossible to differentiate responders form non responders because of the minimal HBsAg decline observed in responders. All HBV genotype A and B infected patients showed a decline of serum HBsAg levels during treatment, with a more pronounced decline in responders. On the contrary, in genotype D patients the decline occurred only in responders, as HBsAg levels remained steady or increased slightly in non-responders [49]. Accordingly, the absence of any HBsAg decline combined with <2 log IU/mL HBV-DNA reduction after 12 weeks of Peg-IFN-α identifies HBV genotype D infected non responders with high accuracy and 100% NPV, providing an effective stopping role to discontinue treatment in 20% of the patients [50]. 

In HBeAg positive, HBV genotype A and D infected patients the lack of any HBsAg decline from baseline to week 12 was the most accurate predictor of non-response (NPV 97–100%), whereas HBsAg levels >20,000 IU/mL were more accurate non-response predictors in HBV genotypes B and C infected patients (NPV 92–98%) [51]. Consequently, HBsAg genotype-specific algorithms had been designed to warrant response-guided therapy.

During antiviral therapy, an intrinsic limitation of constitutive HBV markers (antigens and nucleic acids) is that their kinetics may no longer reflect the virus/host interplay, but result from the direct antiviral activity of the drug, being just the hallmark of target engagement. Overcoming this limit requires new markers more directly expressing the virus/host interplay, accordingly MicroRNAs (miRNAs) profiling had been investigated. miRNAs are small endogenous single-stranded RNAs that modulate the expression of cellular genes and play an important role not only in the cellular homeostasis but also may influence the outcome of both infections and diseases. Cellular miRNAs are released in serum and in circulating HBsAg-particles [52], with highly significant over-expression of miR-122-5p, miR-99a-5p and miR-192-5p in chronic hepatitis patients, as compared to carriers of HBeAg negative infection. The same miRNAs expression progressively declines during antiviral therapy, reaching levels comparable to that observed in HBeAg negative infection at the end of therapy in long-term responders [53]. A combination of the most significant miRNAs (MiR-B-Index) accurately distinguished active from inactive HBV infection and identified patients who achieved a sustained control of HBV infection after Peg-IFN-α. Following studies that confirmed that circulating miRNAs are associated with HBeAg status, levels of HBV DNA and HBsAg, and treatment response [54], identifying miR-3960 as a candidate predictor for HBsAg clearance after PEG-IFNα-2a [55]. More recently, Nagura et al. analyzed 61 CHB patients treated with PEG-IFNα-2a weekly for 48 weeks, of whom 12 had a virological response (VR: HBV-DNA < 2000 IU/mL off therapy for 48 weeks), confirming that pre-treatment serum miR-192-5p levels were significantly correlated with the levels of HBsAg, HBV DNA and hepatitis B core-related antigen [56]. Interestingly, lower miR-192-5p levels at baseline were the only independent factor correlated with VR, and their levels at 24 weeks of treatment remained significantly higher in non-VR patients. All together these findings support the use of miR-192-5p levels to predict therapeutic efficacy of Peg-IFN-α in CHB patients, although larger cohort studies and more standardized assays are required before clinical application.

### 2.2. Nucleos(t)ide Analogues

Nucleos(t)ide Analogues (NAs) target HBV polymerase, an enzyme catalyzing RNA- and DNA-dependent DNA polymerase with both RNase H and protein priming activities (Figure 2). NAs, incorporated into newly synthetized DNA cause chain termination blocking reverse transcription from pre-genome (pg-RNA) to HBV-DNA and strongly inhibiting HBV replication. Additional mechanisms, such as the inhibition of the first or second step of HBV protein priming by competing with dGTP or dAMP by entecavir and tenofovir could further contribute to the antiviral activities [57,58]. Nevertheless, the inhibition of viral replication is not complete and a residual virion production is maintained with possible infection of new hepatocytes. Accordingly, studies in liver humanized mice, showed that during NA treatment, the add-on of the virus entry inhibitor Myrcludex-B increases the antiviral activity blocking the intrahepatic viral spread to uninfected cells and new infections in cells already harboring viral cccDNA [59]. Such evidence supports the hypothesis that in long-term NAs-treated patients, the intercellular spreading of residual infective virions can contribute to the cccDNA pool maintenance. In addition, the residual replicative activity could contribute to persistence of the intracellular cccDNA amplification by which the relaxed circular is directed to the host cell nucleus instead of being secreted in new virions [60]. In fact, NAs do not act on cccDNA neither altering its stability nor inhibiting its transcriptional activity and consequently viral pre-genome and proteins continue to be produced as shown by the divergent HBV-RNA and HBV-DNA kinetics and by the very slow decline of HBsAg, HBeAg and HBcrAg serum levels [61,62].

In spite of the fact that NAs do not directly modulate the immune system activity nor reduce viral antigens production, immunological studies showed that long-term treatment with NAs is accompanied by an improvement of both innate and adaptive immune responses [63]. In NAs-treated patients who cleared HBsAg, the in vitro expansion of HBV-specific T cells showed efficient responses comparable to those of patients who spontaneously recovered from acute hepatitis B. Interestingly, in patients who were HBV DNA negative but still HBsAg positive T-cell responses were lower than in those who cleared HBsAg, but greater than in untreated CHB patients [15]. A working hypothesis is that during NA treatment a modulation of NK-phenotype could contribute to more efficient HBV-specific T-cell responses [64]. 

In summary, long-term NAs treatment, in spite of simply inhibiting viral polymerase, appears to have a quite complex impact on HBV infection. In fact, the reduction of HBV-DNA synthesis results in the decline of hepatocytes new infections, dilution of the intrahepatic cccDNA burden and down-modulation of its transcription: all together these events are responsible for the slow, but progressive decline of circulating viral markers and immune response modulation, that prompts the achievement of the off-therapy HBV infection control, at least in a small proportion of cases [65,66].

#### 2.2.1. Efficacy of NAs Treatment

Lamivudine (LMV), adefovir dipivoxil (ADV), entecavir (ETV), telbivudine (TBV), tenofovir disoproxil fumarate (TDF) and tenofovir alafenamide (TAF) are the currently available NAs and those with potent antiviral activity and high barrier to resistance (ETV, TDF and TAF) are recommended as first line treatment in CHB patients [1,2,3]. 

The antiviral efficacy of NAs was proven since the first availability of LMV that was shown to inhibit viral replication in a significant proportion of both HBeAg positive and negative patients. However, a major limitation of using LMV was posed by the progressive emergence of resistant viral strains in over 70% of patients after >5 years treatment which caused viral breakthroughs usually associated with hepatitis flares, eventually leading to liver failure and death in patients with cirrhosis [67,68]. The emergence of antiviral resistant variants results from the selection of specific mutations in the reverse transcriptase domains of the HBV polymerase gene, which modify the viral polymerase–drug interaction interfering with its inhibitory effect. Furthermore, after the emergence of primary resistance, compensatory mutations may restore a full replication capacity of the virus and the accumulation of secondary resistance mutations increases drug resistance. Antiviral resistant mutants are archived in the cccDNA and persist in the virus population after their selection even if treatment is stopped causing cross-resistance in case of sequential monotherapy [69,70]. Thus, the first line treatment should be a high barrier resistance NA, such as ETV, TDF or TAF, while TDF or TAF have to be used as rescue therapy in case of viral breakthrough to nucleoside analogues [1,2,3]. The virologic and biochemical response rates after 1 year treatment with ETV, TDF and TAF [71,72,73] are reported in Table 1.

After 5 years of ETV or TDF treatment, 99–97% of HBeAg positive naïve CHB patients achieve virologic response (undetectable HBV-DNA), 53–49% HBeAg loss, with anti-HBe seroconversion in 75–81% and HBsAg loss in 10% of them. In HBeAg negative patients the 3 years treatment undetectable HBV-DNA rate is >90% and the HBsAg clearance rate is about 1%/year [74,75,76]. In phase 3 clinical trials, the rate of virological response to TAF after 96 weeks of treatment was comparable to that of TDF in both HBeAg positive and negative patients, with a higher rate of ALT normalization (75% vs. 68% in HBeAg pos patients at week 96, and 50% vs. 32% at week 48 in HBeAg negative patients) [77,78]. 

Due to their optimal safety profile, NAs can be used, with the same treatment schedule either in early CHB patients or patients with advanced, decompensated liver disease or liver transplant. Long-term ETV or TDF monotherapy was shown to halt progression of liver disease, by improving necro-inflammation and significantly reducing fibrosis: regression of cirrhosis was reported in 74% of 96 patients after 5 years of TDF treatment; the lack of histological improvement associated with co-factors of liver damage such diabetes, high BMI and persistence of abnormal ALT [79]. Whether a complete regression of the already established cirrhosis is still possible remains debated, nevertheless a long-lasting suppression of liver disease activity may foster a reduction of intrahepatic fibrosis and the remodeling of micronodular to macronodular cirrhosis [80]. Anyhow, NAs treatment in patients with an early stage of decompensation may improve, control or delay the end stage complications. In fact, since NAs have been widely used in CHB patients the need for liver transplantation for end stage cirrhosis complications was significantly reduced [81]. A recent study on a cohort of 1951 Caucasian patients on long term NA treatment showed an overall survival comparable to that of the general population: a slightly higher mortality rate was observed in cirrhotic as compared to CHB patients and HCC was the major factor affecting mortality [82]. Accordingly, in spite of a reduction of HCC incidence in long-term NA-treated patients, they have to be maintained on HCC surveillance programs [1,2,3], and several scores have been developed for the identification of patients at higher risk [83].

**Table 1:** Response 6 months after the end of 48–52 weeks of Peg-IFN-α and after 48–52 weeks of NA treatment.

Treatment	HBeAg Status (BL)	HBeAg/Anti-HBe SC	HBV-DNA <60–80 IU/mL	ALT Normalization	HBsAg Loss	References
Peg-IFN-α 2a 180 ug qw	Positive	32%	14%	41%	3%	[34,35]
Peg-IFN-α 2b 100 ug qw	Positive	29%	7%	32%	7%	[36]
Peg-IFN-α 2a 180 ug qw	Negative	na	19%	59%	4%	[37]
ETV 0.5 mg qd	Positive	21%	67%	68%	2%	[72]
ETV 0.5 mg qd	Negative	na	90%	78%	0%	[73]
TDF 245 mg qd	Positive	21%	76%	68%	3%	[74]
TDF 245 mg qd	Negative	na	93%	76%	0%	[74]
TAF 25 mg qd	Positive	10%	64%	72%	1%	[78]
TAF 25 mg qd	Negative	na	94%	83%	0%	[79]

BL: baseline; SC: sero-conversion; Peg-IFN-α: pegylated interferon; qw: quoad week; qd: quoad die; ETV: entecavir; TDF: tenofovir dipovixil; TAF: tenofovir alafenamide.

#### 2.2.2. NA Discontinuation

In HBeAg positive CHB patients without cirrhosis NAs can be discontinued in case of negative serum HBV-DNA and HBeAg/anti-HBe seroconversion after a consolidation period of 6–12 months, since anti-HBe seroconversion was shown to be maintained in the majority of patients (>85% 2 years after NAs discontinuation) together with virologic response (HBV-DNA < 2000–20,000 IU/mL) in about 50% at 3 years [1,84]. The duration of the consolidation period (< or >12 months) does not appear to influence the virological relapse rate, occurring mainly within the first 2 years after treatment discontinuation. HBsAg loss is currently the virological end point considered to warrant a safe NAs discontinuation in HBeAg negative CHB patients [1,2,3]. However, the on-treatment HBsAg clearance rate is quite low and in recent years the number of studies on NA discontinuation had been increasing, mainly in Asian patients [85,86,87]. Overall, available data indicate that serum HBV-DNA becomes detectable in most of the patients within the first 6 months after NAs discontinuation, reaching levels >2000 IU/mL in about 50% and 70% of cases at 6 and 12 months respectively. The evidence of clinical relapse (usually defined by HBV-DNA > 2000 IU/mL and abnormal/2 times ULN ALT levels) increases progressively during the first 2 years after NAs discontinuation: in a report where 130 Caucasian and Asian patients were studied, the relapse rates were 35% and 55% at 6 months and 2 years, respectively, with 22% and 40% of patients retreated [87]. Interestingly, a study where 166 German HBeAg negative CHB patients were randomized to maintain treatment or discontinue NAs after 4 years of virological suppression, showed at week 96 that only 14% of the patients restarted treatment, while 31% had HBV-DNA < 20 IU/mL and 10% cleared HBsAg. In this cohort, HBsAg serum levels at the time of NA discontinuation predicted significantly the HBsAg loss which occurred in 28% of patients with HBsAg < 1000 IU/mL and 1.9% of those with HBsAg > 1000 IU/mL [88]. Thus, HBsAg serum levels are currently the best predictor of sustained virological response after NAs discontinuation. The threshold of 100 IU/mL HBsAg serum levels had been proposed as a cutoff to identify patients with low (9–19%) or high (31–87%) risk of clinical relapse [89]. Unfortunately, the proportion of patients who achieve HBsAg serum levels <100 IU/mL, at least in HBV genotype A and D patients, is quite low even after prolonged treatment: therefore, there is an unmet need of algorithms combining different biomarkers or their kinetics for a better prediction of hepatitis B relapse risk and a more effective tailoring of viral suppression duration in the single patient. 

In the setting of NAs treatment where cccDNA transcription is not directly inhibited by therapy, the kinetics of viral markers reflecting its activity, such as HBcrAg and HBV-RNA, could contribute to a better understanding of the virus/host equilibrium dynamics, mainly after a persistent serum HBV-DNA clearance. Accordingly, in a cohort of 62 patients (50 HBeAg positive) HBV-RNA and HBV-DNA strongly correlated at baseline, but their kinetics differed during treatment, with a slower decline of HBV-RNA, that if present between 3–6 months was the strongest predictor of HBeAg to antiHBe seroconversion [90]. Similar to HBV-RNA, HBcrAg serum levels also showed a slower reduction as compared to HBV-DNA: in a cohort of 66 HBeAg negative CHB patients after 3–5 years of NAs, HBcrAg was still detectable in 33–27% and HBV-RNA in 30–14% of cases, respectively, and their presence at NAs discontinuation associated with severe hepatitis B flares [91]. In 572 CHB patients (17% HBeAg positive) who discontinued NAs after a mean treatment period of 295 weeks, lower HBcrAg serum levels associated with higher rate of virological response (OR 0.71, *p* < 0.001), with HBsAg loss (OR 0.476, *p* < 0.001), and lower rates of flares (OR 1.288, *p* = 0.005) [92]. Interestingly the figures were similar for HBsAg serum levels, suggesting that HBsAg decline during NA treatment indeed reflect the achievement of an effective control of HBV infection. Accordingly, a recent paper, where the outcome of NAs discontinuation was studied in 1216 CHB patients, confirmed that HBsAg serum levels < 100 IU/mL is a strong predictor of HBsAg loss and its combination with undetectable HBcrAg further improved the prediction. Interestingly, the study also showed as the patient’s ethnicity (Asian vs. non-Asian) and the genotype (B vs. A, D and C) significantly influenced the probability of HBsAg loss [93]. 

Given the lack of strong predictors of sustained virological response and the possibility of severe and life-threatening hepatitis B reactivations after NAs discontinuation the current EASL CPG suggest in HBeAg negative CHB to consider to stop NAs treatment only in patients without cirrhosis, with at least 3 years of virological suppression and who can be followed closely with ALT and HBV DNA determinations at least during the first year following NAs cessation [1].

## 3. Peg-IFN-α and NAs Combination, Add on and Sequential Therapies

The idea to take advantages from the different mechanisms of action of IFN-α and NAs to achieve an effective and sustained control of HBV infection was pursued since the availability of Lamivudine. Different strategies were attempted, combining the 2 drugs at treatment start, switching from one to the other (sequential therapy) or adding one after monotherapy with the other (add on), but at present none were proven to consistently improve the overall response rate and none of them are currently recommended by international guidelines [1,2,3]. 

As far as it concerns Peg-IFN-α and NAs combination, a randomized trial on 740 CHB patients showed that the Peg-IFN-α and TDF combination for 48 weeks achieved a significantly higher rate of HBsAg clearance (9%) as compared to 16 weeks combination (2.8%) or monotherapy (0% with TDF, 2.8% with Peg-IFN), both in HBeAg positive and negative patients. However, these findings stem mainly from a significantly higher HBsAg clearance rate (about 20%) in HBV genotype A infected patients, who appears to therefore specifically benefit from this approach [94]. The Peg-IFN-α add-on in HBeAg positive patients after 6 months of ETV monotherapy induced a higher HBeAg/anti-HBe seroconversion rate at 48 weeks as compared to monotherapy, and HBsAg serum levels <4000 IU/mL and HBV-DNA < 50 IU/mL associated with a higher response rate [95,96]. However, the extended follow-up of a subset of patients showed that at week 96 the seroconversion rate was similar in combo and monotherapy groups [97], suggesting that Peg-IFN-α anticipates the seroconversion. HBsAg serum levels declined more significantly in long term NAs-treated HBeAg negative patients who combined Peg-IFN-α for 12 months as compared to those who maintained NAs monotherapy; unfortunately, the impact of IFN treatment on NA discontinuation was not investigated in these studies [98,99]. A recent study in 201 CHB patients initially randomized to receive a total of 192 weeks TDF, either in monotherapy or after initial Peg-IFN-α combination for 24 weeks and then re-randomized to maintain or discontinue TDF, showed a rate of HBsAg loss significantly higher in those discontinuing TDF than in those remaining on treatment (6.9 vs. 2.8%), but the results were not influenced by IFN treatment [100]. Whether longer Peg-IFN treatment could influence the achievement of a functional cure remains to be investigated.

Actually, the overall results suggest a potential benefit of the NA/Peg-IFN combination, however there is not yet a single strategy proven adequate for all the patients and key information and criteria for selecting both patients and treatment schedules are still missing. Future studies should correlate the different responses with the virologic, immunologic and clinical features of the patients, to identify biomarkers profiles and effective treatment algorithms that allow for more accurate therapy individualization. 

## 4. Towards Personalized Medicine in CHB

In the near future antiviral treatment for CHB will be enforced by new molecules designed to act on specific viral and immunological targets (Figure 1). At least for the moment, a “sterilizing cure” with the elimination of any HBV trace seems difficult to be obtained, while a reasonable objective appears to be that of switching the interplay with the virus in favor of the host [5]. A consistent reduction of the burden of infected hepatocytes, the shutdown of both cccDNA and integrated DNA transcription and the restoration of a competent immune response should lead to a durable HBsAg loss and serum HBV-DNA clearance. Actually, the functional cure of CHB is already obtained in 10 to 20% of IFN-α treated patients and in a smaller proportion (<10%) of patients on long term NAs, although recent reports suggest that HBsAg loss increases after NA discontinuation [1,2,3,65,85,86,87,88]. Thus, the goal of new therapeutic approaches will be the achievement of functional cure in a significantly higher number of patients [5]. 

It is likely that NAs will remain the backbone of the new therapies either because many patients will be already on treatment or because NAs will be part of the new therapeutic combination, as shown by most of the ongoing Phase 2 studies [101,102,103]. In this perspective it will be advisable to better characterize patients on NAs who, in spite of being all virologically suppressed, have a highly variable control of HBV infection as shown by the different outcomes observed upon treatment discontinuation. To this purpose, on treatment monitoring algorithms combining viral and immunological biomarkers and modelling the dynamics of HBV infection could contribute to better describe virus/host interplay. Furthermore, an improved patient profiling will help to optimize the use of new immunomodulatory molecules, which should ameliorate the immunomodulatory activity of IFN-α, avoiding its adverse reactions. The new drugs, designed to act on specific steps of innate or adaptive immune response and to maximize their efficacy, should be given according to specific individual conditions of the virus/host equilibrium. In absence of an individualized profiling, the multifaceted activity of Peg-IFN-α remains worth to be exploited. In fact, Peg-IFN-α was shown to accelerate the HBsAg decline significantly when given in combination with silencing RNA(VIR 2218): a week 24 serum HBsAg decline ≤ 10 IU/mL was seen in 54.5% of the patients receiving VIR-2218 concurrently with PEG-IFN-α and 13.3% of those receiving VIR-2218 alone [104].

**Figure 3:**
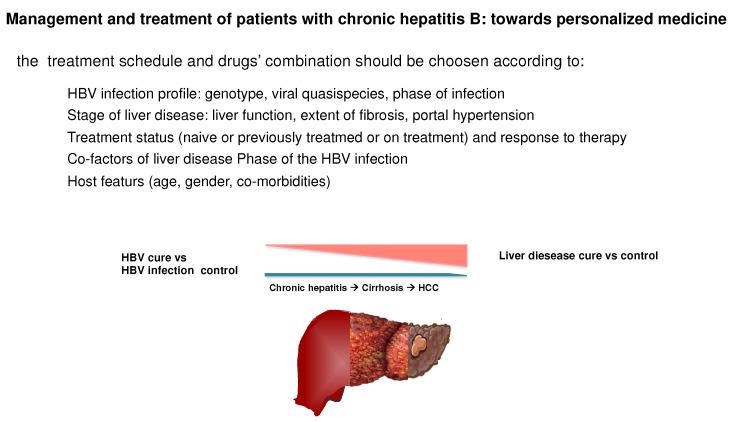
Virologic and clinical characteristics, that should guide the therapeutic choice in chronic hepatitis B patients to grant a personalized approach with both the currently available and the new drugs. The functional cure with a finite treatment should be pursued primarily in young patients with chronic hepatitis, whereas in older patients with cirrhosis the persistent inhibition of both viral replication and disease activity is a reasonable goal of antiviral therapy, as it was shown to stop/reduce the progression to end stage liver disease complications. An accurate characterization of the HBV infection profile and of its evolution overtime, together with the treatment history of the patient will further guide the selection for each patient of the most appropriate treatment strategy, particularly when new antiviral and immune modulatory drugs will be available.

In conclusion, in the near future an effective treatment personalization will be required to fully exploit the potential of the larger spectrum of new treatments. A more accurate definition of the virologic profile in light of the individual treatment history will guide the most appropriate therapeutic choice (Figure 3). However, the decision whether to pursue an off-treatment functional cure or a pharmacological suppression of viral replication will be mainly guided by the severity of liver disease, age and co-morbidities. Accordingly, in more complex and fragile patients NAs therapy will remain the safer and more cost-effective treatment.
